# Physical and mental demands of work associated with dementia risk in later life

**DOI:** 10.1016/j.tjpad.2025.100084

**Published:** 2025-02-02

**Authors:** Hang-Ju Yang, Yun-Chieh Yang, Chih-Cheng Hsu, Wan-Ju Cheng

**Affiliations:** a Department of Emergency Medicine, Jen-Ai Hospital Dali Branch, Taichung, Taiwan; b National Center for Geriatrics and Welfare Research, National Health Research Institutes, Yunlin, Taiwan; c Institute of Population Health Sciences, National Health Research Institutes, Miaoli, Taiwan; d Department of Psychiatry, China Medical University Hospital, Taichung, Taiwan; e Department of Public Health, China Medical University, Taichung, Taiwan

**Keywords:** Cognitive functions, Job control, Job strain, Physical activity, Psychological demands

## Abstract

**Background:**

Work occupies a significant portion of adult life, and both cognitive stimulation and physical activity have been suggested as factors that may lower dementia risk in later life.

**Objectives:**

To examine the association between mental and physical demands at work and the risk of dementia.

**Design:**

A cohort study.

**Setting:**

Seven selected districts in Taiwan, covering both urban and rural areas.

**Participants:**

4,083 community-dwelling healthy adults aged 55 and older from the Healthy Aging Longitudinal Study.

**Measurements:**

A job matrix of work conditions by occupation was generated using data from a representative national survey. Mental demands were assessed by job control and psychological demands from the Job Content Questionnaire, as well as skill levels. Physical demands were assessed using a 4-point Likert scale and dichotomized into high and low levels. Dementia diagnoses were identified based on physician diagnosis registered in the National Health Insurance database.

**Results:**

Over a follow-up period of 6.2 years, 513 participants were diagnosed with dementia. After adjusting for confounding factors in cox regression models, high (vs. low) job control, high -skilled jobs (vs. low), and high physical demands (vs. low) were associated with a reduced future risk of dementia. Psychological demands were not associated with dementia risk.

**Conclusions:**

Greater utilization of job skills and engagement in physically demanding activities at work may help mitigate the risk of developing dementia. The effects of different dimensions of psychological demands on cognitive health warrant further investigation.

## Highlights

• Association of work conditions with diagnosis of dementia in a longitudinal cohort.
• High job control and high-skilled jobs were linked to lower dementia risk.
• Physical but not psychological demands are associated with lower dementia risks.

## 1 Introduction

Enhancing cognitively stimulating activities and physical activities in midlife is recommended as an action to reduce dementia risk [1]. While work takes up a majority of waking hours in adult lives for decades, it acts as an important setting for enhancing cognitive reserve and dementia prevention [2]. The effect of cognitive stimulation at work could be more profound than leisure time cognitive activity and short-term interventions [3,4]. While leisure time physical activity is an important protective factor for dementia prevention [5], little is known about how physical demand at work is associated with dementia in later life.

Studies examining how work influences cognitive function and dementia risks in later life conceptualized cognitive stimulation using either skill complexity by occupations or psychosocial work conditions based on Karasek's job-demand-control (JDC) model [6]. In studies utilizing occupational skill complexity, work complexity was classified according to the U.S. Dictionary of Occupational Titles [7], which provides job-title-specific scores from the 1971 Current Population Survey [[8], [9], [10]]. However, given variations in the speed of job automation in different industries [11], skill ratings established 50 years ago may no longer apply to current jobs across different countries. Other scales have been used as well, including skill level based on the International Standard Classification of Occupations 2008 (ISCO-08) [12,13], and the Occupational Information Network (O*NET) rating scale by the U.S. Department of Labor [[14], [15], [16]]. Despite different scales used, findings are consistent that individuals working jobs with higher complexity and skill levels had a cognitive advantage in later life [10,12,[17], [18], [19], [20]]. However, few studies examined the influence on clinically diagnosed dementia.

On the other hand, the JDC model examines job control and psychological demands, with low job control and high psychological demands considered work stressors. Job control encompasses two components: decision latitude, which involves having the power to make decisions, and skill discretion, which pertains to using a variety of skills and learning new things at work. Different combinations of high and low levels of job control and demands correspond to varying levels of work stress. For example, high psychological demands paired with high job control—referred to as “active jobs”—are suggested to enhance cognitive function [21]. In contrast, a high-strain job, characterized by high psychological demands and low job control, is associated with burnout and is detrimental to cognitive function [22]. Notably, psychological demands in the JDC model are intended to capture the stressful psychological burden. It is not clear whether this demand is “protective” or “harmful” to cognitive function.

The health effects of physical activity at work and during leisure time were different. For cardiovascular events and mortality, higher leisure time physical activity is associated with low risk, while higher occupational physical activity is associated with increased or non-significant risks [[23], [24], [25]]. In contrast, other studies observed that moderate occupational physical activity is associated with lower mortality [26], particularly in men [27]. To date, the few studies that have examined the association between occupational physical activity and dementia have had inconsistent findings [28,29]. These studies were also limited by a lack of consideration for mental demands at work.

This study aims to examine the association between mental and physical demands of major work in life and the risk of clinical dementia of different types. To address the limitations of previous studies, we linked the cohort with national health insurance data for registered diagnosis of dementia as outcome. Additionally, we used two different scales, namely scales based on the JDC model and skill levels, to evaluate mental stimulation at work. Our hypotheses are: (1) low job control but not high psychological demands is associated with lower risk of dementia; (2) jobs that require higher skill are associated with lower risk of dementia; and (3) physical demand at work is associated with lower risk of dementia.

## 2 Methods

### 2.1 Study design

This study combined data from the Healthy Aging Longitudinal Study in Taiwan (HALST) cohort, medical claim data from Taiwan's National Health Insurance Research Dataset (NHIRD), and occupational-level work condition data from the national Occupational Health and Safety Survey (OSHS). All data were de-identified before being handed to the authors. This study has been approved by the ethics committee of the National Health Research Institutes (EC1090903).

### 2.2 Study participants

The HALST recruited healthy community-dwellers aged 55 and above in seven selected districts in Taiwan. These seven locations cover both urban and rural areas, as well as different ethnic groups, representing the diverse sociodemographic characteristics of the Taiwanese population [30]. In each catchment area, a regional hospital was selected, and all eligible residents living within a 2-km radius of this local hospital were recruited using a systematic sampling method. Exclusion criteria were bedridden, hearing impairment, self-reported clinical diagnosis of dementia, or too frail to complete the questionnaire or clinical examinations. The first wave survey was conducted since 2009 and the second wave follow-up started in 2014. A total of 5663 and 4232 participants completed the first and second wave survey. Participants were interviewed at home for those who completed the consent form. All 5663 participants were asked to provide consent for linking their data with health claim records in the NHIRD. Taiwan's universal National Health Insurance program, launched in 1995, covers approximately 99 % of the national population [31]. The NHIRD contains detailed medical claim data, including diagnoses, medications, and procedures from both outpatient and inpatient settings.

**Fig. 1:**
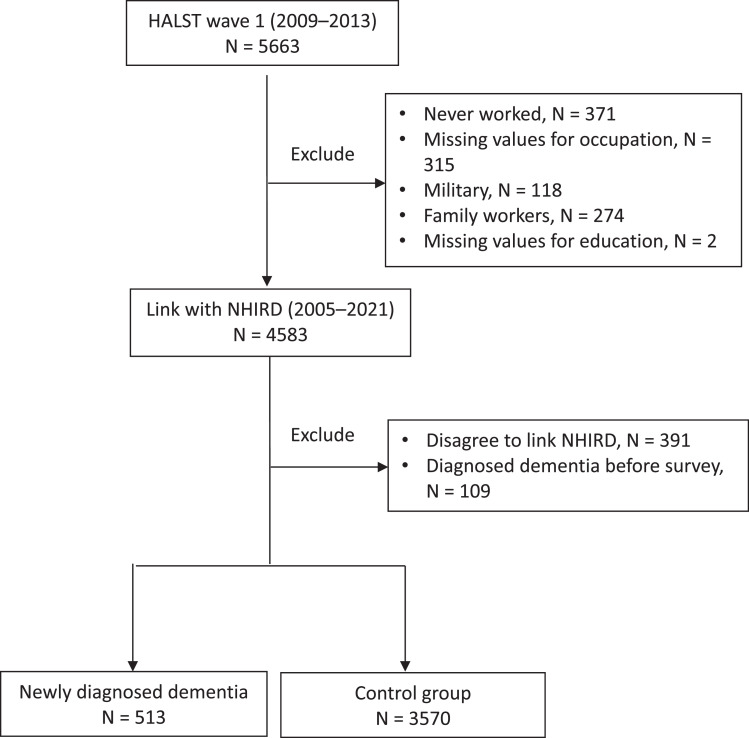
Sample-selection flowchart.

HALST participants were asked about their work history, their major occupation (worked for the longest duration or the main income source) in their lifetime, and whether they were employed at the time of the survey. The exclusion criteria are as follows: participants who reported never having worked in both waves of the survey (N = 371), those with missing information on occupation (N = 315) or education (N = 2), individuals serving in the military (N = 118), family workers (N = 274), participants who did not consent to have their data linked to the NHIRD (N = 391), and those with a prior diagnosis of dementia in the NHIRD at the time of the HALST survey (N = 109) (Fig. 1).

Due to the lack of individually reported work conditions in the HALST, a job matrix was created to identify work conditions by occupation. This matrix was aggregated from the 2016 OSHS by averaging the work condition scores of individuals aged 50 and above. Occupations were self-reported and categorized according to the Standard Occupational Classification by the Ministry of Labor of Taiwan. Occupations in the HALST were also categorized according to the same classification system, as well as the International Standard Classification of Occupations (ISCO-08) two-digit major and sub-major groups, enabling occupation-level work conditions to be linked with HALST data. The job exposure matrix, which connects job titles with work factors, has been widely used for assessing work exposures [32]. Although it does not account for individual heterogeneity within the same job title, it provides an efficient method to incorporate workplace exposures that are often missing in health cohort studies.

The OSHS, conducted by the Ministry of Labor of Taiwan, has been administered to a nationwide working population every three to five years since 1988, with the goal of gaining insights into workplace safety and health conditions. For each survey of the OSHS, participants were selected using a two-stage random sampling process. Individuals who were currently employed at the time of the survey were identified from the sampled households. The interviews were conducted face-to-face by trained interviewers. The sample's representativeness was carefully ensured, and detailed information regarding the survey's sampling methodology can be found elsewhere [33].

### 2.3 Outcome measurement

Diagnosis of dementia following the HALST survey date was identified from the NHIRD for HALST participants by diagnostic codes ICD-9-CM code 290.0–290.4, 290.8–290.9, 331.0), and was categorized into Alzheimer's dementia (ICD-9-CM 331.0), vascular dementia (ICD-9-CM 290.40–290.43), and other dementia (ICD-9-CM 290.0, 290.10–290.13, 290.20–290.21, 290.3, 290.8–290.9, 291.2). At least two outpatient visits or at least one hospitalization claim were required to be identified as having dementia. Follow-up was censored at the time of death identified in NHIRD or at the end of the NHIRD data (December 2021), whichever came first.

### 2.4 Physical and mental demands at work

Physical demands were assessed with a single item from the OSHS survey: “my job requires a lot of physical effort”. Respondents were asked to indicate their level of agreement on a four-point Likert scale ranging from 1 (strongly disagree) to 4 (strongly agree). Occupations were categorized into high and low physical demands based on the median score (50.7).

For the assessment of mental demands, we included psychological job demands, job control, and the level of job skill utilization. Psychological job demands and job control were assessed using the validated Chinese version of the Job Content Questionnaire (JCQ) based on the JDC model developed by Karasek and Theorell [34,35]. The OSHS questionnaires in 2016 included seven items for the job-control scale (learning new things, non-repetitive work, creative work, freedom to make decisions, various tasks, influential opinions, and the development of one's abilities), as well as five items for psychological job demands (fast work, hard work, concentration on the job for a long time, hectic work, and insufficient manpower). The scores were summed, standardized, and subsequently divided into low and high groups based on the median (50.8 for psychological demands and 51.6 for job control). According to this model, four types of occupations were identified for the job demand-control matrix: (1) high strain: low control and high demands, (2) low strain: high control and low demands, (3) active jobs: high control and high demands, and (4) passive jobs: low control and low demands.

The ISCO-08 classifies jobs into 436 groups at the finest level of detail, with aggregation to 10 major groups, which are mapped onto one of four hierarchical skill levels: skill level 1 (routine physical or manual tasks), skill level 2 (tasks such as operating machinery, maintenance, storage of information), skill level 3 (performance of complex technical and practical tasks), and skill level 4 (complex problem solving, decision-making, and creativity). In this study, we categorize jobs into low (skill level 1), medium (skill level 2), and high (skill levels 3 and 4) work complexity [12].

### 2.5 Covariates

Age, sex, education years, and marital status were self-reported from the HALST. Marital status was reported as married, separated, cohabiting, divorced, widow or widower, and never married, and we grouped the participants into married and otherwise conditions. Body weight and height were obtained during clinical examination, and body-mass index (BMI) was calculated as body weight (kg) divided by square of body height (m). Missing values for BMI were imputed using mean values.

Regarding health behaviors, we included smoking, drinking behavior, and leisure time activity. Smoking status was assessed through participant self-reporting of current smoking habits, categorized as either yes or no. Heavy drinking was identified as drinking more than 14 drinks in men and 7 drinks in women per week. Participants were asked about specific exercises they performed during the previous year. The recorded activities were converted to metabolic equivalent task (MET)-minutes. For each participant, the mean total energy consumption in kcals per week was calculated as the sum of the MET score of the specific physical activity multiplied by the frequency and duration in the past two weeks and body weight on average [36]. Missing values for METs were imputed using mean values.

History of major physical illness was assessed by the Charlson Comorbidity Index (CCI) from diagnostic codes in the NHIRD. The CCI is a widely used indicator of medical comorbidities [37], will be employed to represent physical health based on 19 diagnoses excluding dementia (i.e., myocardial infarction, heart failure, peripheral vascular disease, cerebrovascular disease, chronic pulmonary disease, rheumatologic disease, peptic ulcer disease, mild liver disease, hemiplegia or paraplegia, renal disease, malignancy, moderate or severe liver disease, metastatic solid tumor, acquired immune deficiency syndrome, leukemia, lymphoma, and diabetes with and without chronic complication) in the NHIRD from 2005 to the HALST recruitment year. Participants were categorized into three groups by CCI score 0, 1, and ≥ 2.

Mental disorders were identified using diagnostic codes, including mood disorders (ICD-9-CM codes 296, 300, 311) and psychotic disorders (ICD-9-CM codes 295, 297) in the NHIRD from 2005 until the HALST recruitment year. Participants were divided into two groups based on the presence or absence of any mental disorder.

Other work conditions in the OSHS surveys included a seven-item scale for the assessment of workplace justice (trust, reliable information, fair work arrangements, fair rewards, fair performance evaluation, inclusion of information in decision-making, and respect). This scale was modified from the original standard questionnaire and demonstrated good psychometric properties [38]. One item was used to assess job insecurity (“my job is insecure”). Responses for these items were recorded on a four-point Likert scale, ranging from one (strongly disagree) to four (strongly agree). The scores were summed, standardized, and then divided into low and high groups based on the median (59.9 for workplace justice 48.5 for and job insecurity).

### 2.6 Statistical analysis

Chi-square tests were used to compare sociodemographic characteristics and work conditions between participants who developed dementia and the control group. The association between mental and physical work demands and dementia was examined using Cox proportional hazard models. The Cox models were adjusted for gender, education level, marital status, cigarette smoking, heavy drinking, exercise behaviors, body mass index, Charlson comorbidity index, work status at the time of the HALST survey, job insecurity, and workplace justice. Multicollinearity among the covariates was tested, and all showed a variance inflation factor of less than 6 (Supplementary Table 1). The model assumptions were tested using the Schoenfeld residuals test. Age was treated as the time scale in the model, using age at baseline and age at the event or censoring. The model assumptions were tested using the Schoenfeld residuals test. To account for mortality as a competing risk factor in the relationship between work conditions and the risk of dementia, we conducted a sensitivity analysis using the subdistribution hazard function. The interaction between gender and work conditions in relation to dementia risk was also examined. Kaplan–Meier survival curves of the four types of jobs according to the job demand-control matrix were illustrated using the two-tailed log-rank test. We further identified different types of dementia and examined their association with mental and physical work demands, respectively. Data analysis was performed using SAS version 9.4 (SAS Institute, Cary, NC, USA).

## 3 Results

Among the 4083 participants, 513 cases (12.6 %) of incident dementia were observed during the follow-up period of an average 6.2 years (Table 1). Compared to the control group, those who developed dementia were older (74.1 vs. 68.0 years old), had lower education, lower household income, higher comorbidity index scores, and a higher percentage of mental disorders. Regarding work conditions of their major jobs, a higher proportion of the dementia group reported low job control (59.1 % vs. 49.3 %), passive jobs (35.3 % vs. 28.7 %), and low or medium job skill level. Work conditions differed significantly by gender and education level (Supplementary Table 2). A higher proportion of men reported low physical and psychological demands, high job control, and high job skill levels compared to women. Participants with longer years of education more frequently reported low physical job demands, but higher psychological demands, greater job control, and higher job skill levels compared to those with fewer years of education.

**Table 1:** Sociodemographic characteristics of study participants at wave 1 survey (N = 4083).

	Dementia	Control	
	(*n* = 513)	(*n* = 3570)	
Characteristics	N (%)	N (%)	*p*
Age (years)			< 0.001
55–64	44 (8.6)	1311 (36.7)	
65–74	243 (47.4)	1576 (44.2)	
≥ 75	226 (44.0)	683 (19.1)	
Education years			< 0.001
< 6	137 (26.71)	595 (16.67)	
6	173 (33.72)	1169 (32.75)	
7–12	132 (25.73)	1117 (31.29)	
≥ 13	71 (13.84)	689 (19.3)	
Gender (female)	240 (46.8)	1529 (42.8)	0.101
Marital status (married)	349 (68.0)	2735 (76.6)	< 0.001
Cigarette smoking (yes)	70 (13.7)	549 (15.4)	0.338
Heavy drinking (yes)	20 (3.9)	255 (7.1)	0.008
Body mass index ≥ 30 kg/m2	32 (6.2)	219 (6.1)	1.000
Charlson comorbidity index			< 0.001
0	86 (16.8)	1090 (30.5)	
1	132 (25.7)	924 (25.9)	
≥ 2	295 (57.5)	1556 (43.6)	
Mental disorders (yes)	176 (34.3)	828 (23.2)	< 0.001
Household income			< 0.001
High	39 (7.6)	614 (17.2)	
Low	263 (51.3)	1470 (41.2)	
Missing	211 (41.1)	1486 (41.6)	
Working at the time of survey	103 (20.1)	1268 (35.5)	< 0.001
Physical job demands			0.102
High	276 (53.8)	1779 (49.8)	
Low	237 (46.2)	1791 (50.2)	
Psychological demands			0.720
High	249 (48.5)	1767 (49.5)	
Low	264 (51.5)	1803 (50.5)	
Job control			< 0.001
High	210 (40.9)	1811 (50.7)	
Low	303 (59.1)	1759 (49.3)	
Job demand-control matrix			< 0.001
Passive jobs	181 (35.3)	1024 (28.7)	
Low-strain jobs	83 (16.1)	779 (21.8)	
High-strain jobs	122 (23.8)	735 (20.6)	
Active jobs	127 (24.8)	1032 (28.9)	
Job skill level			0.043
Low	45 (8.8)	269 (7.5)	
Medium	326 (63.5)	2119 (59.4)	
High	142 (27.7)	1182 (33.1)	
Job insecurity			0.019
High	277 (54)	1726 (48.3)	
Low	236 (46)	1844 (51.7)	
Workplace justice			0.011
High	232 (45.2)	1834 (51.4)	
Low	281 (54.8)	1736 (48.6)	
			
	Mean (SD)	Mean (SD)	*p*
Age (years)	74.13 (7.12)	67.96 (7.88)	< 0.001
Education (years)	7.04 (4.90)	8.49 (4.79)	< 0.001
Follow-up duration (years)	6.20 (3.29)	10.04 (2.68)	< 0.001
Leisure time activity (METs)	929 (1356)	1010 (1417)	0.379

*Abbreviations:* METs = metabolic equivalents.

^⁎^P values comparing the two groups were calculated using chi-square tests and the Mann-Whitney test.

The Schoenfeld residuals test did not reveal a violation of the proportional hazards assumption, as all p-values were > 0.05 (Supplementary Table 3). After adjusting for all covariates (Table 2), psychological demands were not associated with dementia risk. High job control was associated with a 0.7-fold hazard of developing dementia compared to low job control (HR = 0.70, 95 % confidence interval [CI] = 0.57 to 0.85). High skilled-jobs were associated with a 0.6-fold hazard of developing dementia compared to low-skilled jobs (HR = 0.61, 95 % CI = 0.39 to 0.95), and high-physical-demand jobs were associated with a 0.6-fold hazard of dementia compared to low-physical-demand jobs (HR = 0.63, 95 % CI = 0.43 to 0.94). The interaction between gender and work conditions was not significant. In the sensitivity analysis using the subdistribution hazard function, where mortality was identified as a competing risk factor, the results were consistent with the primary findings (Supplementary Table 4).

**Table 2:** Associations between mental and physical demands at work and the risk of dementia incidence (N = 4083).

Work condition	Model 1	Model 2	Model 3	
	Crude HR (95 % CI)	Adj HR (95 %CI)	Adj HR (95 %CI)	P value for interaction with gender
Physical demands (high vs. low)	1.09 (0.91–1.29)	0.72 (0.48–1.09)	0.63 (0.43–0.94)*	0.503
Psychological demands (high vs. low)	1.03 (0.87–1.23)	1.13 (0.93–1.36)		
Job control (high vs. low)	0.69 (0.58–0.82)⁎⁎⁎	0.70 (0.57–0.85)⁎⁎⁎		
				
Job skill level				0.218
Low	Reference		Reference	
Medium	0.81 (0.59–1.11)		0.79 (0.57–1.10)	
High	0.68 (0.48–0.95)*		0.61 (0.39–0.95)*	

*Abbreviations:* HR = hazard ratio; 95 % CI = 95 % confidence interval.

⁎ p <  0.05; ** p <  0.01;.

⁎⁎⁎ p <  0.001. ^†^Model 1 was a univariate Cox proportional hazard model. Models 2 and 3 were adjusted for gender, education years, marital status, household income, cigarette smoking, heavy drinking, metabolic equivalents, body mass index, Charlson comorbidity index, mental disorders, work status at survey, job insecurity, and workplace justice.

**Fig. 2:**
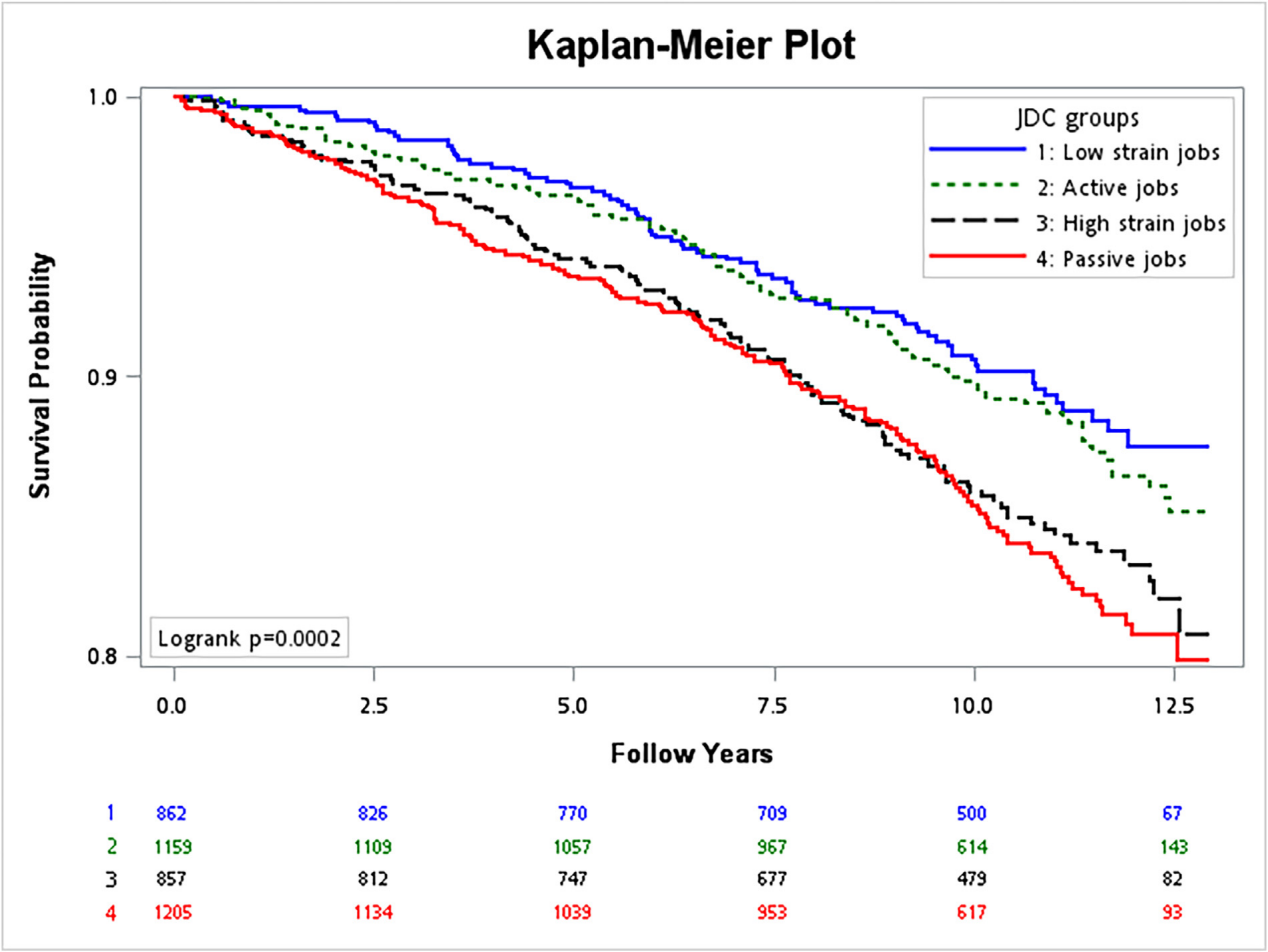
The Kaplan-Meier survival curves for dementia based on the job demand-control (JDC) matrix groups. The total follow-up duration was 39,041 person-years.

When occupations were categorized into four groups using the job demand-control matrix, Fig. 2 revealed that high-strain and passive jobs were associated with a lower survival probability for developing dementia compared to low-strain and active jobs. After adjustment for confounders, high-strain and passive jobs were associated with 1.61- and 1.45-fold hazards of developing dementia compared to low-strain jobs (Supplementary Table 5).

We further examined the risk for different types of dementia, namely Alzheimer's dementia, vascular dementia, and other type dementia (Table 3). High-physical-demand jobs were associated with a lower risk of dementia types other than Alzheimer's and vascular dementia (adjusted HR = 0.62, 95 % CI = 0.39 to 0.97). When follow-up duration, rather than age, was used as the time scale (Supplementary Table 6), high-physical-demand jobs were associated with a lower risk of Alzheimer's dementia (adjusted HR = 0.38, 95 % CI = 0.15 to 0.97) and other types of dementia (adjusted HR = 0.47, 95 % CI = 0.29 to 0.76) but not vascular dementia.

**Table 3:** Associations between mental and physical demands and the risk of different types of dementia (N = 4083).

Work demands	Alzheimer's dementia (Case = 101)	Vascular dementia (Case = 99)	Other dementia‡ (Case = 432)
	Adj HR (95 %CI)	Adj HR (95 %CI)	Adj HR (95 %CI)
Physical demands (high vs. low)	0.55 (0.23–1.31)	1.98 (0.82–4.77)	0.62 (0.39–0.97)*
Psychological demands (high vs. low)	1.02 (0.66–1.56)	0.94 (0.61–1.47)	1.15 (0.93–1.42)

*Abbreviations:* HR = hazard ratio; 95 % CI = 95 % confidence interval.

⁎ p <  0.05. ^†^All models were adjusted for gender, education years, marital status, household income, cigarette smoking, heavy drinking, metabolic equivalents, body mass index, Charlson comorbidity index, mental disorders, work status at survey, job insecurity, workplace justice, skill discretion, and decision authority.

‡ Other dementia include ICD-9-CM 290.0 Senile dementia, uncomplicated; 290.10 Presenile dementia uncomplicated; 290.11 Presenile dementia with delirium; 290.12 Presenile dementia with delusional features; 290.13 Presenile dementia with depressive features; 290.20 Senile dementia with delusional features; 290.21 Senile dementia with depressive features; 290.3 Senile dementia with delirium; 290.8 Other specified senile psychotic conditions; 290.9 Unspecified senile psychotic condition; and 291.2 Other alcoholic dementia.

## 4 Discussion

This is the first study to examine the risk of developing clinical dementia in older age based on different levels of work-related mental demands, assessed using both the JDC model and job skill level. High job control was associated with decreased future risk of dementia compared to low job control, and high-skilled jobs were associated with decreased future risk of dementia compared to low-skilled jobs. Furthermore, a novel finding is that physical demands, but not psychological demands, were associated with a lower risk of dementia.

Our finding that high-skilled jobs and high job control are associated with a lower risk of dementia is consistent with previous studies showing similar associations with dementia [39] and cognitive functions as the outcome [13,15,40,41]. Older individuals retired from occupations characterized by higher complexity and skill have cognitive advantage in later life [10,12,[17], [18], [19], [20]]. For specific cognitive function, low job control was negatively associated with executive function, psychomotor speed, phonemic fluency, and semantic fluency after retirement. Passive and high-strain jobs, both of which were characterized by low job control, were associated with lower scores on phonemic and semantic fluency when compared to low-strain jobs [42]. Possible mechanisms linking psychosocial work conditions and cognitive function include telomere length, inflammation, and protein related to axonogenesis and synaptogenesis in the central nervous system [21,43]. While evidence supports that cognitive stimulation at work offers protection against neurodegeneration, job-strain models have also shown a relationship with dementia.

Regarding the job demand-control matrix, one study used diagnosed dementia as the outcome and found that active jobs were associated with a lower risk of dementia compared to passive jobs [21]. Similar findings were observed in studies using cognitive scales as the outcome, where passive jobs and high-strain jobs were associated with cognitive decline [40,44]. In older adults specifically, active jobs were associated with a slower rate of disability progression [45,46]. Our findings are consistent in that passive jobs showed an elevated risk of dementia, but we suggest that job control, rather than psychological demands, was the key element associated with dementia risk rather than psychological demands.

In the current literature, findings regarding psychological demands at work and cognitive function are inconsistent. Two studies, which included both manual and non-manual workers, similar to the participants in this study, found no association [42,47]. Nevertheless, one study of white-collar workers found an association between low psychological demands and cognitive decline [40]. One possible explanation is that the original nine-item scale for psychological demands encompasses various aspects of workload, including time pressure, work volume, and intense concentration. As a result, the psychological-demands scale may reflect a combination of stress and cognitive stimulation, which could lead to mixed effects on dementia risk, particularly among manual workers. Further investigation into the effects of different dimensions of psychological demands on cognitive health is warranted. For instance, studies examining occupational-based mental demands have shown that demands related to language and knowledge, pattern detection, information processing, and service are associated with a slower rate of cognitive decline [16,48]. Additionally, jobs that involve interaction with people have shown the strongest evidence for a reduced risk of Alzheimer's disease, compared to jobs that involve working with inanimate objects [8]. Therefore, psychological demands may have varying cognitive effects in manual versus non-manual workers.

Our findings on the beneficial effect of physical demands on dementia risk are novel but differ from previous studies, which showed either no association or an increased risk of work-related physical activity with dementia [28,29]. One possible explanation is that we adjusted for mental demands, which may have confounded the association in earlier studies. Another distinction is that the previous studies measured physical activity at work during the 1970s and 1980s, when physical demands were likely higher than in modern workplaces. The introduction of automation across industries have significantly reduced the physical demands, particularly in jobs with heavy physical demands [49]. Therefore, our findings may reflect the beneficial effects of physical activity in modern workplaces, where sedentariness at work is a threat to cognitive health [50]. Meanwhile, because intense physical activity at work is related to less physical activity in leisure time [51,52], we have adjusted leisure time METs in our model. The effect of physical demands at work remained significant, possibly due to leisure activities becoming increasingly sedentary in modern society [53]. Low physically demanding work, for example brain work in a seated position, promotes glycemic instability and hypercortisolemia, which can affect energy metabolism and body composition [54]. In this study, the association was most significant for Alzheimer's dementia. Possible biological mechanisms include physical-activity-related increases in neurotrophic factors, hippocampal neurogenesis, modulation of inflammation, and reductions in oxidative stress and the amyloidogenic pathway [55]. Nevertheless, the lack of a significant association between physical demands and Alzheimer's dementia in the Cox models using age as the time scale suggests that age is a major confounding factor in this relationship. The lack of association between physical demands at work and vascular dementia is likely due to the strict adjustment for cardiovascular and cerebrovascular diseases in the statistical model.

This study is strengthened by using a longitudinal cohort and clinically validated dementia. Furthermore, the use of two types of measurement for mental demands confirmed the hypothesis that use of skills, but not the psychological burden of work, showed protective effects for cognitive health in older ages. Nevertheless, this study has limitations. First, the HALST recruited healthy community-dwelling individuals older than 55 years; therefore, selection bias limits the generalization of the study results. We excluded individuals who developed dementia at younger ages and lacked data on the retirement age of our participants. Early exit from the labor market may lead to a reduced influence of work conditions on dementia risk in later life. Second, the occupation-based work conditions from the JDC model were sourced from a 2016 national survey, while participants’ working years likely spanned from 1980 to 2010, during which work conditions may have changed. Trends indicated a decline in job control and increasing job demands from 2001 to 2016 [56], particularly for high-skilled workers. As a result, the true level of job control may have been underestimated for participants in high-skilled jobs. In this case, using job-control data from 2016 may have weakened the observed association between job control and dementia. Additionally, with the trend toward job automation, physical demands have shifted, such as an increase in exposure to awkward positions [49]. In this study, using a single item to evaluate physical demands obscured the complexity of these demands. Third, some individual-level confounders were not considered in this study. For example, the job matrix omitted individual variability in perceived physical and mental demands within occupations. These may include factors such as an individual's stress coping abilities, different work positions, and varying organizations within the same occupation. Furthermore, ApoE4 amplifies the impact of passive jobs and low mental demands of work on dementia and cognitive decline [15,57]. Although ApoE4 is unlikely to be a confounder that associates with the exposure (i.e., work conditions) in this study, it could moderate the association between work conditions and dementia risk. Future studies should include genetic variables to better understand the individual differences that affect the outcomes.

In conclusion, our work suggests that high physical demands, high skill level used at work, and high job control are associated with lower risk of developing dementia. Work conditions could serve as a protective, modifiable ecological factor for preventing dementia at the organizational level. Interventions to improve job control and skill used include participative technique of quality circles, work reorganization, facilitating two-way communication between management and employees, commitment and cooperation of the managers, and conflict-management skills [58]. Job control can be shared as a group perception, and enhancing job control showed positive effects on absenteeism and productivity [59]. More studies are needed to explore specific elements of organizational-level interventions that modify cognitive health in later life. Furthermore, a sedentary pattern of work is increasing even among blue-collar workers [60]. Organizational-level intervention targeting increasing physical activity at work includes height-adjustable desks, activity-based working, and scheduled physical breaks for white-collar workers, as well as physical exercises such as stretching at the beginning and end of a workday for blue-collar workers [61,62].

## Funding

This work was supported by the 10.13039/501100004737National Health Research Institutes [grant number 12A1-CGGP06–051]. The sponsors had no role in the design and conduct of the study; in the collection, analysis, and interpretation of data; in the preparation of the manuscript; or in the review or approval of the manuscript.

## CRediT authorship contribution statement

**Hang-Ju Yang:** Writing – original draft. **Yun-Chieh Yang:** Formal analysis. **Chih-Cheng Hsu:** Data curation. **Wan-Ju Cheng:** Writing – review & editing, Conceptualization.

## Conflict of interest

The authors declare that they have no known competing financial interests or personal relationships that could have appeared to influence the work reported in this paper.

## Appendix Supplementary materials

Image, application 1
