# Supplementary material for: Physical and mental demands of work associated with dementia risk in later life
Source: J Prev Alzheimers Dis. 2025 Feb 2;12(4):100084. doi: 10.1016/j.tjpad.2025.100084 (PMC12183963; doi:10.1016/j.tjpad.2025.100084)
Supplement: Supplementary file 1 [file mmc1.docx]

Supplementary Table 1. Multicollinearity among the covariates included in the Cox proportional hazards model.

| Variables | Variance inflation factor |
| --- | --- |
| Physical job demands | 5.173 |
| Psychological demands | 1.173 |
| Job control | 1.260 |
| Age | 1.341 |
| Gender | 1.457 |
| Education years | 1.931 |
| Marital status | 1.161 |
| Household income |  |
| Low | 2.603 |
| Missing | 2.479 |
| Cigarette smoking | 1.216 |
| Heavy drinking | 1.086 |
| Leisure time activity (METs) | 1.094 |
| Body mass index ≥ 30 kg/m^2^ | 1.019 |
| Charlson comorbidity index |  |
| 1 | 1.434 |
| ≥ 2 | 1.519 |
| Mental disorders | 1.057 |
| Working at the time of survey | 1.238 |
| Job insecurity | 4.807 |
| Workplace justice | 5.674 |

Supplementary table 2. Work conditions of participants by sex and educational level.

|  | Gender | | |  | Education years | | | | |
| --- | --- | --- | --- | --- | --- | --- | --- | --- | --- |
|  | Men | Women | p |  | <6 years | 6 years | 7-12 years | ≥ 13 years | p |
|  | N (%) | N (%) |  |  | N (%) | N (%) | N (%) | N (%) |  |
| Physical job demands |  |  | <0.001 |  |  |  |  |  | <0.001 |
| High | 1092 (47.19) | 963 (54.44) |  |  | 598 (81.69) | 954 (71.09) | 442 (35.39) | 61 (8.03) |  |
| Low | 1222 (52.81) | 806 (45.56) |  |  | 134 (18.31) | 388 (28.91) | 807 (64.61) | 699 (91.97) |  |
| Psychological demands |  |  | <0.001 |  |  |  |  |  | <0.001 |
| High | 1347 (58.21) | 669 (37.82) |  |  | 238 (32.51) | 603 (44.93) | 598 (47.88) | 577 (75.92) |  |
| Low | 967 (41.79) | 1100 (62.18) |  |  | 494 (67.49) | 739 (55.07) | 651 (52.12) | 183 (24.08) |  |
| Job control |  |  | <0.001 |  |  |  |  |  | <0.001 |
| High | 1253 (54.15) | 768 (43.41) |  |  | 254 (34.70) | 488 (36.36) | 622 (49.80) | 657 (86.45) |  |
| Low | 1061 (45.85) | 1001 (56.59) |  |  | 478 (65.30) | 854 (63.64) | 627 (50.20) | 103 (13.55) |  |
| Job demand-control matrix |  |  | <0.001 |  |  |  |  |  | <0.001 |
| Passive jobs | 515 (22.26) | 690 (39.01) |  |  | 284 (38.80) | 433 (32.27) | 411 (32.91) | 77 (10.13) |  |
| Low-strain jobs | 452 (19.53) | 410 (23.18) |  |  | 210 (28.69) | 306 (22.80) | 240 (19.22) | 106 (13.95) |  |
| High-strain jobs | 546 (23.60) | 311 (17.58) |  |  | 194 (26.50) | 421 (31.37) | 216 (17.29) | 26 (3.42) |  |
| Active jobs | 801 (34.62) | 358 (20.24) |  |  | 44 (6.01) | 182 (13.56) | 382 (30.58) | 551 (72.50) |  |
| Job skill level |  |  | <0.001 |  |  |  |  |  | <0.001 |
| Low | 132 (5.7) | 182 (10.29) |  |  | 115 (15.71) | 136 (10.13) | 57 (4.56) | 6 (0.79) |  |
| Medium | 1293 (55.88) | 1152 (65.12) |  |  | 570 (77.87) | 1017 (75.78) | 729 (58.37) | 129 (16.97) |  |
| High | 889 (38.42) | 435 (24.59) |  |  | 47 (6.42) | 189 (14.08) | 463 (37.07) | 625 (82.24) |  |

P-values are based on chi-square tests comparing work conditions across gender and education groups.

Supplementary Table 3. Results of the Schoenfeld residuals test for the Cox proportional hazards model.

| Variables | P value |
| --- | --- |
| Physical job demands | 0.787 |
| Psychological demands | 0.208 |
| Job control | 0.141 |
| Gender | 0.775 |
| Education years | 0.054 |
| Marital status | 0.205 |
| Household income |  |
| Low | 0.872 |
| Missing | 0.093 |
| Cigarette smoking | 0.727 |
| Heavy drinking | 0.571 |
| Leisure time activity (METs) | 0.941 |
| Body mass index ≥ 30 kg/m^2^ | 0.781 |
| Charlson comorbidity index |  |
| 1 | 0.186 |
| ≥ 2 | 0.609 |
| Mental disorders | 0.419 |
| Working at the time of survey | 0.792 |
| Job insecurity | 0.608 |
| Workplace justice | 0.437 |

Supplementary Table 4. Associations between mental and physical demands at work and the risk of dementia incidence, analyzed using the sub-distribution hazard function (N = 4083).

| Work condition | Model 1 |  | Model 2 |  | Model 3 |
| --- | --- | --- | --- | --- | --- |
|  | Crude SHR (95% CI) |  | Adj SHR (95%CI) |  | Adj SHR (95%CI) |
| Physical demands (high vs. low) | 1.01 (0.85–1.21) |  | 0.64 (0.41–0.99)* |  | 0.58 (0.38–0.89)* |
| Psychological demands (high vs. low) | 1.02 (0.86–1.21) |  | 1.12 (0.92–1.36) |  |  |
| Job control (high vs. low) | 0.71 (0.60–0.85)*** |  | 0.73 (0.60–0.89)** |  |  |
|  |  |  |  |  |  |
| Job skill level |  |  |  |  |  |
| Low | Reference |  |  |  | Reference |
| Medium | 0.86 (0.62–1.18) |  |  |  | 0.85 (0.61–1.18) |
| High | 0.76 (0.54–1.17) |  |  |  | 0.70 (0.45–1.09) |

*Abbreviations:* SHR = sub-distribution hazard ratio; 95% CI = 95% confidence interval.

* p < 0.05; ** p < 0.01.

^†^Model 1 was a univariate Cox proportional hazard model. Models 2 and 3 were adjusted for gender, education level, marital status, household income, cigarette smoking, heavy drinking, metabolic equivalents, body mass index, Charlson comorbidity index, mental disorders, work status at survey, job insecurity, and workplace justice.

Supplementary Table 5. Associations between different types of jobs according to the job demand-control matrix and the risk of dementia (n=4083).

| Job demand-control matrix | Adj HR (95%CI) | p |
| --- | --- | --- |
| Low-strain jobs (low demands + high control) | Reference | - |
| Active jobs (high demands + high control) | 1.15 (0.81–1.61) | 0.435 |
| Passive jobs (low demands + low control) | 1.45 (1.10–1.92) | 0.009 |
| High strain jobs (high demands + low control) | 1.61 (1.20–2.16) | 0.001 |

^＊^All models were adjusted for gender, education level, marital status, household income, cigarette smoking, heavy drinking, metabolic equivalents, body mass index, Charlson comorbidity index, mental disorders, work status at survey, physical job demands, job insecurity, and workplace justice.

Supplementary Table 6. Associations between mental and physical demands and the risk of different types of dementia (N = 4083).

| Work demands |  | Alzheimer’s dementia  (Case = 101) |  | Vascular dementia  (Case = 99) |  | Other dementia^‡^  (Case = 432) |
| --- | --- | --- | --- | --- | --- | --- |
|  |  | Adj HR (95%CI) |  | Adj HR (95%CI) |  | Adj HR (95%CI) |
| Physical demands (high vs. low) |  | 0.38 (0.15–0.97)* |  | 1.64 (0.67–4.03) |  | 0.47 (0.29–0.75)** |
| Psychological demands (high vs. low) |  | 0.95 (0.62–1.46) |  | 0.90 (0.58–1.39) |  | 1.07 (0.87–1.32) |

*Abbreviations:* HR = hazard ratio; 95% CI = 95% confidence interval.

* p < 0.05; ** p < 0.01.

^†^All models used follow-up duration as time scale, and were adjusted for age, gender, education years, marital status, household income, cigarette smoking, heavy drinking, metabolic equivalents, body mass index, Charlson comorbidity index, mental disorders, work status at survey, job insecurity, workplace justice, skill discretion, and decision authority.
